# Proteomics-Compatible Fourier Transform Isotopic Ratio Mass Spectrometry of Polypeptides

**DOI:** 10.1021/acs.analchem.2c03119

**Published:** 2022-10-17

**Authors:** Hassan Gharibi, Alexey L. Chernobrovkin, Amir Ata Saei, Xuepei Zhang, Massimiliano Gaetani, Alexander A. Makarov, Roman A. Zubarev

**Affiliations:** † Division of Physiological Chemistry I, Department of Medical Biochemistry and Biophysics, Karolinska Institutet, Stockholm171 77, Sweden; ‡ Pelago Bioscience, Solna171 65, Sweden; § Department of Cell Biology, Harvard Medical School, Boston, Massachusetts02115, United States; ∥ Chemical Proteomics, Department of Medical Biochemistry and Biophysics, Karolinska Institutet, Stockholm171 77, Sweden; ⊥ Unit of Chemical Proteomics, Science for Life Laboratory (SciLifeLab), Stockholm171 77, Sweden; # Thermo Fisher Scientific GmbH, Dreieich, Bremen28199, Germany; ∇ Department of Pharmacological & Technological Chemistry, I.M. Sechenov First Moscow State Medical University, Moscow119146, Russia; ○ The National Medical Research Center for Endocrinology, 115478Moscow, Russia

## Abstract

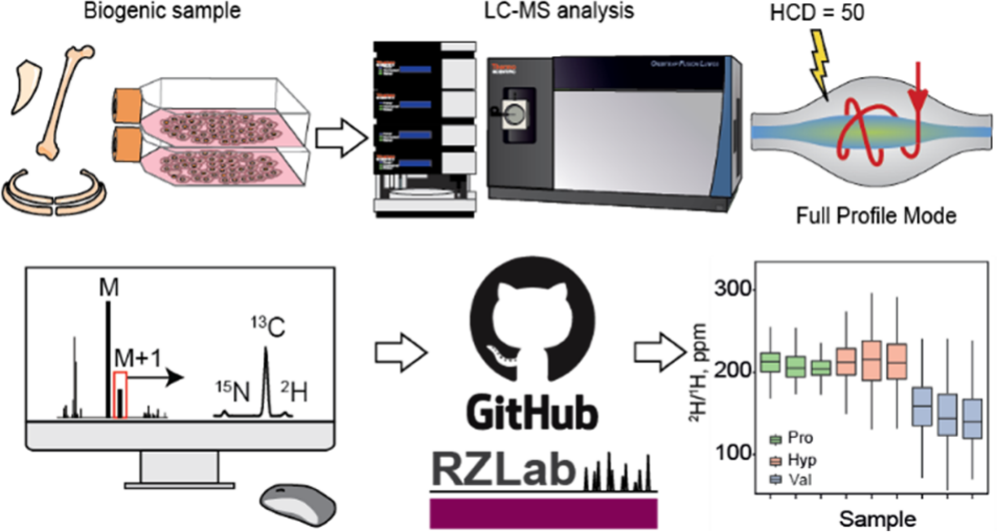

Measuring the relative abundances of heavy stable isotopes of the elements C, H, N, and O in proteins is of interest in environmental science, archeology, zoology, medicine, and other fields. The isotopic abundance measurements of the fine structure of immonium ions with ultrahigh resolution mass spectrometry obtained in gas-phase fragmentation of polypeptides have previously uncovered anomalous deuterium enrichment in (hydroxy)proline of bone collagen in marine mammals. Here, we provide a detailed description and validation of this approach and demonstrate per mil-range precision of isotopic ratio measurements in aliphatic residues from proteins and cell lysates. The analysis consists of proteomics-type experiment demanding sub-microgram amounts of a protein sample and providing concomitantly protein sequence data allowing one to verify sample purity and establish its identity. A novel software tool protein amino acid-resolved isotopic ratio mass spectrometry (PAIR-MS) is presented for extracting isotopic ratio data from the raw data files acquired on an Orbitrap mass spectrometer.

Most elements in nature have more than one stable isotope, with light elements having heavy isotopes as minor components, e.g., carbon (the relative abundance of ^13^C is normally 1.1%), nitrogen (^15^N, 0.37%), hydrogen (^2^H, 150 parts per million, ppm), and oxygen (^18^O, 0.2%).^1^ The isotopic compositions of these biologically important elements vary in nature due to chemical, physical, and biological fractionation occurring in the environment^2^ or individual organism.^3^ Investigation of the stable isotope abundances is frequently used in different areas of science, such as archeology,^4^ zoology,^5^ forensics,^6^ environmental science,^7,8^ and medicine,^9,10^ as well as industrial research for confirming the origin of organic chemicals,^11^ food products,^12^ and in other fields,^13−15^ for example, for obtaining insights into the evolution of ecological environment.^16^

Different elements are involved in specific biochemical processes to a different degree, and thus, some isotopes reflect a given process better than others. For instance, hydrogen isotope analysis is used for modeling and reconstructing the diet of a specimen,^13^ while the ^18^O/^16^O ratios are used to study the migration pattern of species.^17^ As there is a positive correlation between the trophic level and enrichment in heavy isotopes of carbon and nitrogen,^18,19^ measuring the relative amounts of ^13^C and ^15^N is one of the routine ways to analyze specific food chains in nature. The correlation of ^18^O and ^2^H enrichments in water and ice helped researchers to build in the 1970s a model for interpretation of global climate changes.^20^

Isotopic ratio mass spectrometry (conventional abbreviations are IR MS or IRMS, but here we prefer IsoR MS to avoid confusion with Fourier transform infrared spectroscopy) measures the ratio of stable isotopes using, as a rule, magnetic sector mass spectrometers. The results are usually presented as a deviation δ from a standard in ‰ (per mil); e.g., for hydrogen,
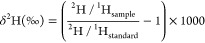
While widely used in practice, IsoR MS has certain shortcomings, of which a major one is the large sample amount requirements: in routine analysis, the rule of thumb is that at least 1 mg of a purified material is needed for measuring each isotope.^21^ This is a critical limitation when it comes to precious samples, such as archeological findings, e.g., animal or human bone fragments. Typically, bone’s yield of collagen ranges between 5 and 10%,^22^ while older bones yield less due to mineralization. Furthermore, the collagen extracted from older bones could be contaminated by proteins from microorganisms or fungi. The bulk sample analysis in IsoR MS cannot differentiate between collagen and other proteins, which can lead to an interpretation error. Another problem in IsoR MS arises from the lack of amino acid resolution when analyzing protein samples. Since not all of the amino acids are essential for a given organism, interpretation of isotopic ratios derived from bulk IsoR MS analysis in terms of an organism’s diet could be erroneous.^23^ To address this issue, in the recently developed compound-specific isotope analysis (CSIA) approach, the protein is first hydrolyzed to free amino acids, which then undergoes derivatization to more volatile forms that can be readily separated via gas chromatography, combusted or pyrolyzed to gas, and analyzed in a conventional IsoR mass spectrometer.^23^ Although CSIA solves some of the problems of bulk analysis, measurements of hydrogen isotopes in amino acids remains challenging due to the ^1^H/^2^H exchange of carbon-bonded hydrogens slowly ongoing at hydrolysis conditions of low pH and high temperature. Also, the sample requirements in CSIA remain rather high.^23^

Another general obstacle for wider use of IsoR MS is the highly specific instrumentation that needs extensive calibration and maintenance, as well as special training and skills. At the same time, many modern labs dealing with protein analysis utilize high-resolution Fourier transform (FT) mass spectrometers of either the Orbitrap or ion cyclotron resonance type. In a typical proteomics LC-MS/MS experiment, these instruments produce extensive sequence and abundance information from sub-microgram quantities of a sample. One approach is to use for isotopic measurements, the isotopic envelope of the polypeptide molecular ions;^24^ however, this method does not provide amino acid resolution. Another approach is to perform gas-phase dissociation of polypeptide ions, fragmenting them down to immonium ions and detecting the product ions with high resolution. The abundance of individual components in the fine structure of the M + 1 peak in comparison with the abundance of the monoisotopic M peak will then yield the ^15^N/^14^N, ^13^C/^12^C, and ^2^H/^1^H ratios, while the fine structure of the M + 2 peak of oxygen-containing immonium ions can provide the ^18^O/^16^O ratio.^25^

Previously, we employed this version of FT IsoR MS for analysis of bone collagen in marine mammals and discovered anomalous deuterium enrichment in (hydroxy)prolines of seals and some other animals.^26^ The δ^2^H values found in that study were very high (≥1000‰); these findings were supported by the CSIA-type IsoR MS analysis. Before application of FT IsoR MS to measuring lesser enrichment degrees, a thorough validation of the approach is needed. Here, we provide such a validation and demonstrate per mil-range precision of isotopic ratio measurements for C, H, and N in aliphatic residues from individual proteins (collagen) and cell lysates.

The sample source for FT IsoR MS analysis can be any protein-containing biological material (Figure 1). The proteins need to be extracted and digested by a protease, e.g., trypsin, as is common in proteomics. The FT isoR MS analysis of a thus obtained peptide mixture consists of an experiment that is very similar to that in conventional proteomics: an LC-MS/MS analysis with data-dependent acquisition (DDA) of MS/MS data.^27^ In that experiment the survey MS spectrum is first taken, and the *m*/*z* values of ions tentatively attributed to peptides are identified. Then, several most abundant such ions are automatically selected one by one in a quadrupole mass filter using a narrow-range *m*/*z* window and fragmented by higher-energy collisional dissociation (HCD) into sequence-specific fragment ions with detection in either a high-resolution Orbitrap or low-resolution linear ion trap detector. The information provided allows one to confirm the expected sequence of the analyzed protein and eventually determine the presence of undesirable contaminants. To that experimental sequence of events, a new MS/MS event is added, called here isoMS, in which all precursor ions are selected with a broad-range *m*/*z* window in a data-independent manner. These ions are fragmented using HCD at higher energy that fragments most peptide bonds and leads to formation of immonium ions (protonated amino acid residues minus the carbonyl group). The product ions of the isoMS event are detected with nominal mass resolution ≥50,000 at *m*/*z* 200 in the range from *m*/*z* 50 to 200 that contains all immonium ions except those of glycine (*m*/*z* 30) and alanine (*m*/*z* 44). From the abundances of the monoisotopic peak M and those of the fine structure isotopic peaks of M + 1, relative abundances of the stable isotopes of ^13^C, ^2^H, and ^15^N in a given immonium ion can be calculated by normalizing the area under the corresponding fine structure peak by the area under the monoisotopic peak divided by the number of atoms of a given type in the immonium ion. In δ^2^H measurements, one has to take into account that the ionizing proton as well as hydrogens at heteroatoms are coming from the solvent that usually has a normal deuterium content of 140–150 ppm. For the ^18^O relative abundance, the corresponding fine structure isotopic peak of the M + 2 ions is used. The obtained measurements are usually already close to the final values, but for better accuracy, they have to be corrected by comparing to a well-characterized standard. As no protein standard with precisely known isotopic composition of each amino acid is commercially available, an interim standard can be used.

**Figure 1:**
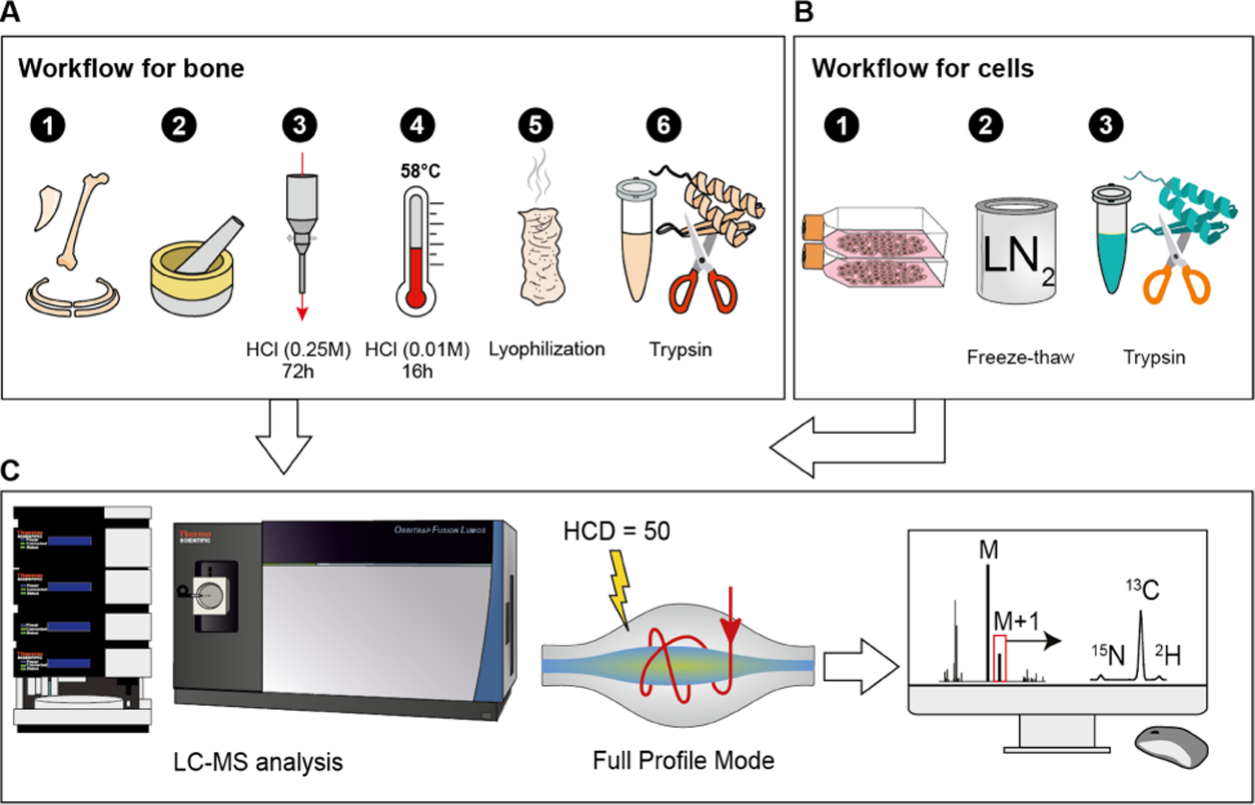
(A) Workflow for bone collagen FT IsoR MS analysis, 1: cleaning bone with deionized water and scraping outer layers, 2: grinding a bone piece into powder, 3: demineralizing bone powder in a glass funnel with 0.25 M HCl for 72 h, 4: gelatinization of collagen with 0.01 M HCl at 58 °C for 16 h, 5: lyophilization of freeze-dried collagen gel overnight, 6: digestion of collagen with trypsin overnight. (B) Workflow for FT IsoR MS analysis of cells, 1: seeding and growing cells in a flask or on a plate, 2: freeze–thaw cycles for cell lysis and protein extraction, 3: digestion of extracted protein with trypsin overnight. (C) Injection of desalted peptides into an LC-MS/MS instrument and FT IsoR MS analysis using a full profile mode and HCD = 50% for peptide ions selected in a broad *m*/*z* window.

The goal of the current study was to determine the type and conditions of the isoMS experiment that provide the highest repeatability of the results and determine the precision of measuring individual isotopes. We also would like to test the FT IsoR MS technique on samples of different types and origins.

## Experimental Section

### Materials

Free amino acids proline (Pro), hydroxyproline (Hyp), and valine (Val) along with ammonium bicarbonate (Ambic), dithiothreitol (DTT), and iodoacetamide (IAA) were purchased from Sigma-Aldrich (St. Louis, MO). Acetonitrile with 0.1% formic acid (v/v) and water with 0.1% formic acid (v/v) (both—LC/MS grade) were purchased from Fisher Scientific. Sequencing grade trypsin was purchased from Promega. Bone samples from different species were collected from the National Museum of Scotland and the Natural Museum of Stockholm. Seed samples were purchased in the form of flour or granules from a local grocery store (oat and coconut flour produced by Risenta AB, corn flour by Molino Favero, Italy, quinoa and millet granules by Go Green AB, Sweden).

### Method

#### Direct Infusion FT IsoR MS

Free amino acids were dissolved in buffer A containing 98% water, 1.9% acetonitrile, and 0.1% formic acid at a 10 μM concentration. Amino acid samples were injected individually using a syringe pump (Hamilton) at a rate of 1 μL/min into an Ion Max electrospray ion source of the Thermo Scientific Orbitrap Fusion Lumos mass spectrometer. Three replicate experiments (*n* = 3) were performed to record the isotopic ratio data; each injection lasted 180 min. The acquisition cycle consisted of one MS event and three follow-up events: a tSIM event, an MS/MS event with HCD = 0, and a targeted MS/MS event with HCD = 50%. The automatic gain control (AGC) target for all events was set at 5e4 with a maximum injection time (IT) of 10 ms. In the MS, tSIM, and MS/MS HCD = 0 events, the molecular ions MH^+^ were recorded (theoretical *m*/*z* for MH+ ion of Pro = 116.0712, Hyp = 132.0661, and Val = 118.0868). The MS/MS event with HCD = 50% produced immonium ions at *m*/*z* 70.0656, 86.0606, and 72.0813, respectively. The targeted MS/MS event selected precursor ions with a charge state of +1 at *m*/*z* 125 with an isolation window of 150 *m*/*z* units wide. The number of microscans per mass spectrum was set at 20. The ion detection was performed with a full profile mode (no noise reduction) at a nominal resolution 60,000 @ 200 *m*/*z*. The detected *m*/*z* range was 50–200 for the targeted MS/MS event and 50–250 for other events.

#### Collagen Samples

Collagenous samples were prepared based on the Brown collagen extraction protocol^28^ as follows. A bone piece of ca. 50 mg was ground to powder, which was then demineralized at room temperature (RT) in 0.25 M HCl for 72 h. The sample was then filtered through glass fiber prefilters (0.7 μm pore size, hydrophilic glass fiber, 25 mm diameter, Millipore), and the insoluble part was incubated in 0.01 M HCl at 58 °C for 16 h to solubilize (gelatinize) the collagen. After freezing the solubilized collagen, samples were freeze-dried (lyophilized) overnight and approximately 100 μg of sponge-like collagens were dissolved in 50 mM Ambic buffer at 70 °C for 3 h. Dissolved collagen was then digested overnight with trypsin (1:50, collagen to trypsin) at 37 °C. Samples were then desalted using a HyperSep Filter Plate C18 (Thermo Fisher Scientific) and dissolved in buffer A to a concentration of 0.5 μg/μL.

#### Cell Lysate Samples

Cell samples were prepared as in the label-free proteomics workflow.^29^ Cells were seeded at a density of 1 million cells per 25 cm^2^ culture flasks. After treatment, cells were detached by trypsinization and washed twice with PBS. Cells were then resuspended in PBS and then underwent five freeze–thaw cycles to disrupt the cell membrane and extract proteins. The extracted proteins were then reduced and alkylated using DTT and IAA, respectively, and then digested with trypsin using the same procedure as for bone collagen. Samples were then desalted and dissolved in buffer A at a 0.5 μg/μL concentration.

#### Plant Seed Samples

Seed samples were ground first and then lysed in a fresh 8M Tris-urea lysis buffer. After the reduction and alkylation step using DTT and IAA, respectively, proteins were precipitated according to the methanol/chloroform protocol.^30^ Samples were then desalted and dissolved in buffer A at a 0.5 μg/μL concentration.

#### LC-FT isoR MS

Thermo Scientific Orbitrap Fusion Lumos and Q Exactive mass spectrometers equipped with an EASY-Spray source and connected online to an UltiMate 3000 RSLC nanoUPLC system were used. The peptide mixture samples were preconcentrated before injection and desalted online using a PepMap C18 nano trap column (length, 2 cm; inner diameter, 75 μm; particle size, 3 μm; pore size, 100 Å; Thermo Fisher Scientific) at a flow rate of 3 μL/min for 5 min. Peptide separation was performed using an EASY-Spray C18 reversed-phase nano LC column (Acclaim PepMap RSLC; length, 50 cm; inner diameter, 75 μm; particle size, 2 μm; pore size, 100 Å; Thermo Fisher Scientific) at 55 °C and a flow rate of 300 nL/min. Peptides were separated using a binary solvent system consisting of 0.1% (v/v) formic acid and 2% (v/v) acetonitrile in water (solvent A) and 98% acetonitrile (v/v) and 0.1% (v/v) formic acid in water (solvent B). For collagen samples, the elution gradient was from 4% B to 15% B for 50 min, increased to 35% B in 10 min and to 95% B in 3 min, stayed at 95% B for 7 min, and then decreased to 4% B in 1 min. For cell lysate samples, the elution gradient was from 4 to 55% B in 55 min, increased to 95% B in 5 min, stayed at 95% B for 5 min, and then decreased to 4% B in 3 min. For seed samples, the elution gradient was 2–22% B in 50 min, increased to 30% B in 10 min, ramped up to 90% in 2 min, stayed at 90% for 5 min, and then decreased to 5% in 1 min.

#### Protein Sequence Identification and Quantification

The protein identification and quantification were based on the DDA method that uses collision-induced dissociation (CID) fragmentation for MS/MS and the linear ion trap for MS/MS ion detection. The *m*/*z* range of MS spectra was set to 375–1500 *m*/*z* in Orbitrap with a resolution of 120,000 @ 200 *m*/*z* and quadrupole isolation. The dynamic exclusion duration was set to 30 s and selection of peptides with charge state of 2–7. For each MS scan, the top 10 precursors were selected for MS/MS fragmentation using CID = 35%, the isolation window of 2 *m*/*z* units in quadrupole and linear ion trap for detection.

#### isoMS Event for Peptide Analysis

Targeted MS/MS was used with the *m*/*z* range for selecting the precursor ions from 300 to 1300, an inclusion list selecting ions at *m*/*z* 800, charge state of 2+, with a 1000 *m*/*z* unit wide isolation window. The selected ions underwent fragmentation with HCD = 50%, and the fragment ions were detected by the Orbitrap analyzer in the *m*/*z* range 50–200 at a nominal resolution of 60,000. Each MS/MS event was acquired with 20 microscans.

#### Isotopic Ratio Determination

**Figure 2:**
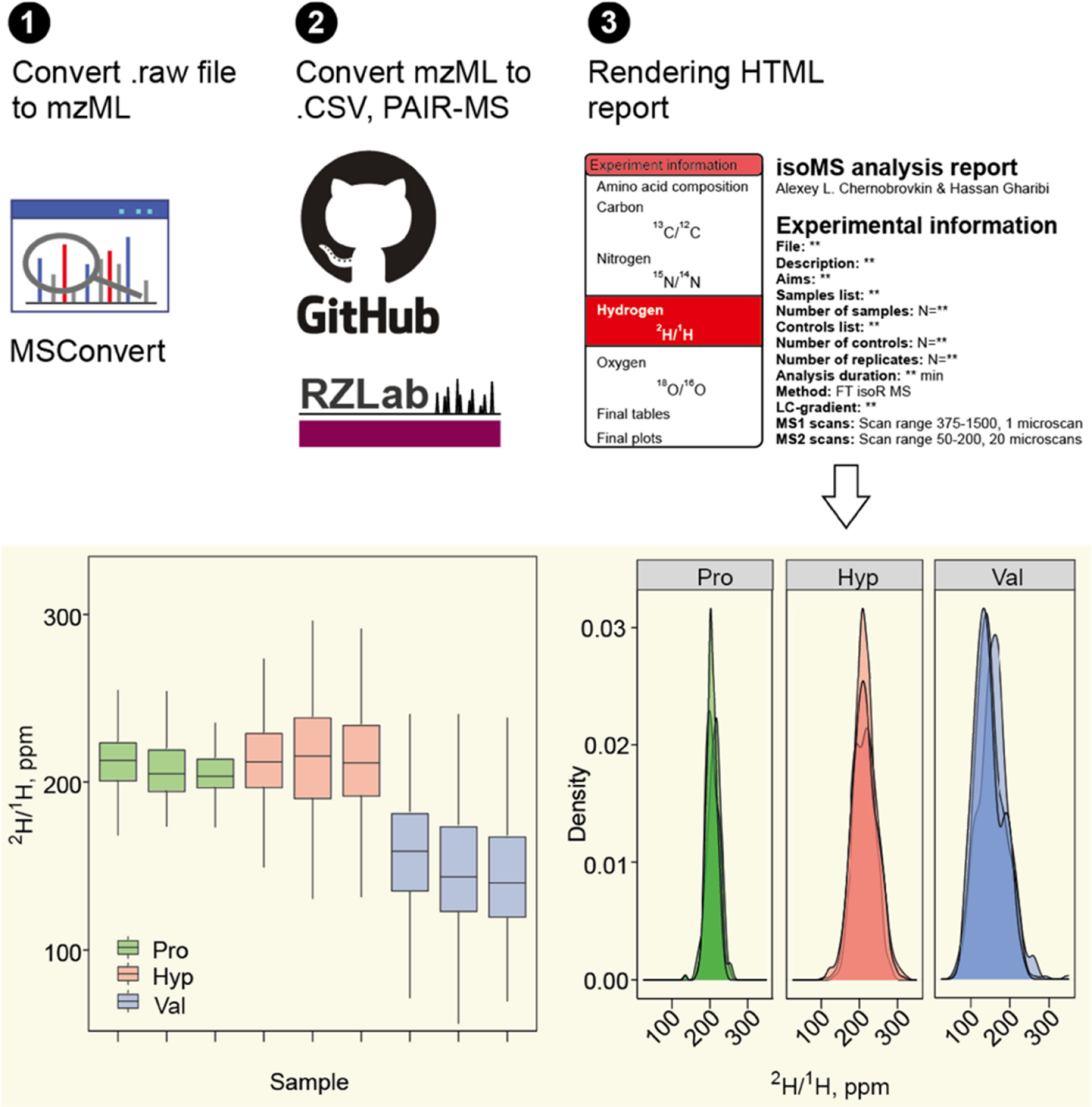
FT IsoR MS data processing consists of three steps. 1: conversion of. raw files to .mzML files using e.g., MSConvert online tool, 2: conversion of .mzML to .csv files using in-house github package PAIR-MS, 3: creating an HTML report from the .csv files using PAIR-MS.

The acquired .raw files were converted to .mzML format using MSConvert (version 3.0.20168) from ProteoWizard. The .mzML files were then reformatted to a .csv table using the in-house PAIR-MS (https://github.com/hassanakthv/PAIR-MS) github package. The isotopic ratios were determined as explained in Gharibi et al.^26^ An “experiment design” .csv file was created for producing the HTML report using the same github package as shown in Figure 2.

## Results

### FT IsoR MS Method Validation—Direct Infusion

**Figure 3:**
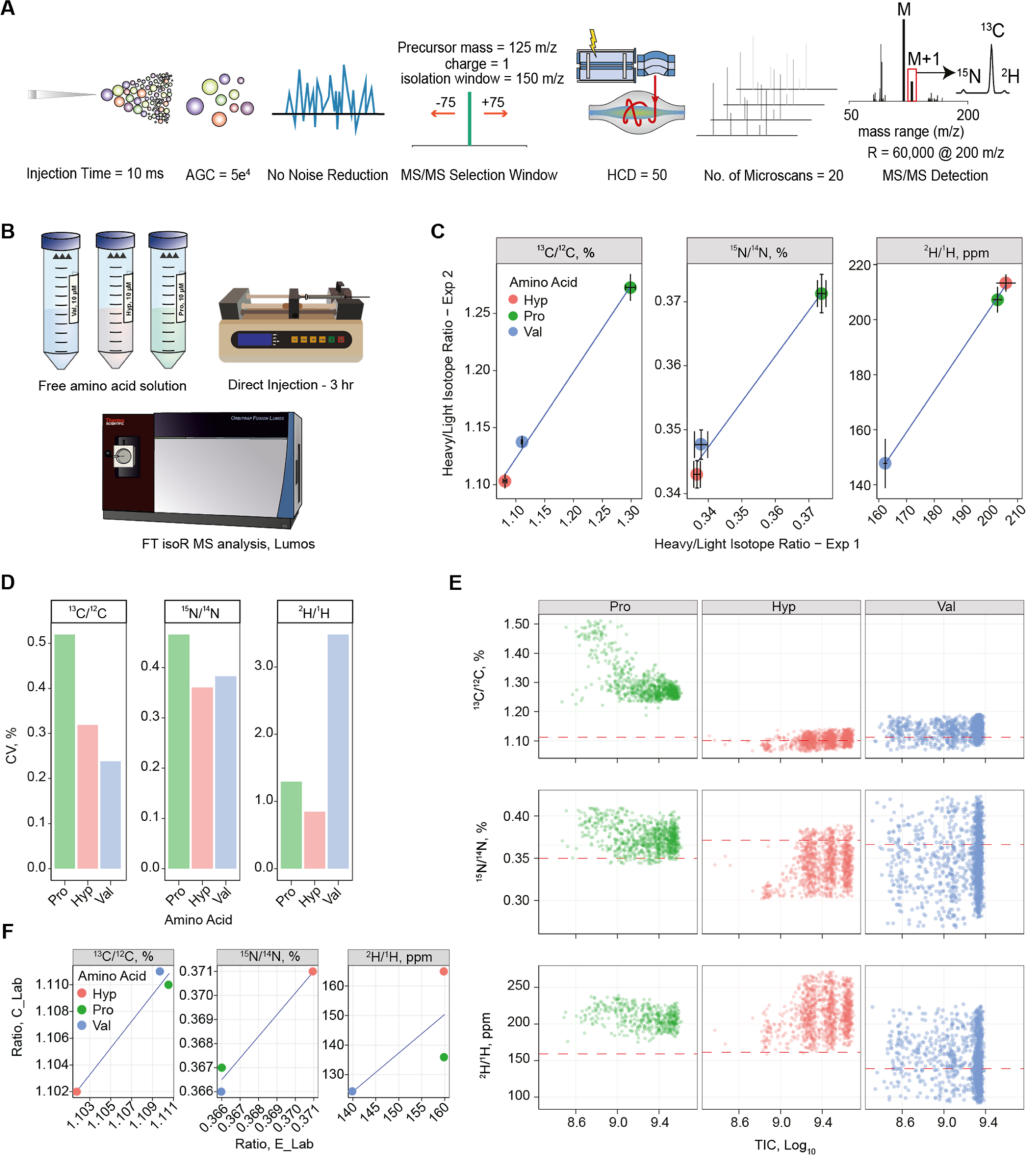
(A) Selected FT IsoR MS parameters for analysis of free amino acids. (B) Free solutions (10 μM) of amino acids Pro, Hyp, and Val were directly infused by a syringe pump into a mass spectrometer. (C) Linear regressions between the FT isoR MS isotopic ratio measurements for C, H, and N in Pro, Hyp, and Val from two independent experiments performed with a 4-week interval. (D) Coefficients of variation (CVs) between the replicate FT isoR MS analyses of each amino acid for C, H, and N. (E) Recorded isotopic ratios vs. total ion current (TIC) in each mass spectrum. The red dashed line denotes the IsoR MS results from the same sample obtained by an external service laboratory (E_Lab). (F) Comparison of the IsoR MS measurements performed on the three amino acid samples by two external service laboratories.

Validation of the FT IsoR MS method was performed on free amino acids Pro, Hyp, and Val because these were available in a pure form and large quantities and could be analyzed for control by two independent international service labs using conventional IsoR MS. The best FT IsoR MS data acquisition approach was identified based on two criteria: (a) repeatability of the results with almost one month in between two independent FT IsoR MS analyses and (b) coefficient of variability (CV) of the isotopic ratios between the technical replicates within the same experiment (amino acids were injected one by one in every replicate). As MS produces stronger ion signal than MS/MS, we expected that the molecular ions MH+ would produce superior isotopic ratio data to the MS/MS-obtained immonium ions. However, targeted MS/MS with HCD = 50% (Figure 3A,B) unexpectedly turned out to be the only technique providing repeatable data (Figure 3C), while the MH+ based approaches failed to reproduce even the order of amino acids in terms of the isotopic ratios.

Targeted MS/MS also provided the lowest CVs (Figure S1), which for ^13^C/^12^C and ^15^N/^14^N ratios were 0.5% or less, and for ^2^H/^1^H, they were below 1% (Figure 3D). Figure 3E depicts the isotopic ratios acquired in each mass spectrum plotted against the log_10_-transformed total ion current. There is no discernible trend, confirming that due to AGC, the measurements did not depend upon the ion current.

Surprisingly, the results from the service laboratories obtained on the same samples were not superior to FT IsoR MS, failing to agree even on the order of amino acids in terms of ^13^C abundances (Figure 3F). It is fair to note that these laboratories performed their routine analyses and were not provided further information on experiment design.

### LC-FT IsoR MS Method Validation—C3 vs C4 plants

**Figure 4:**
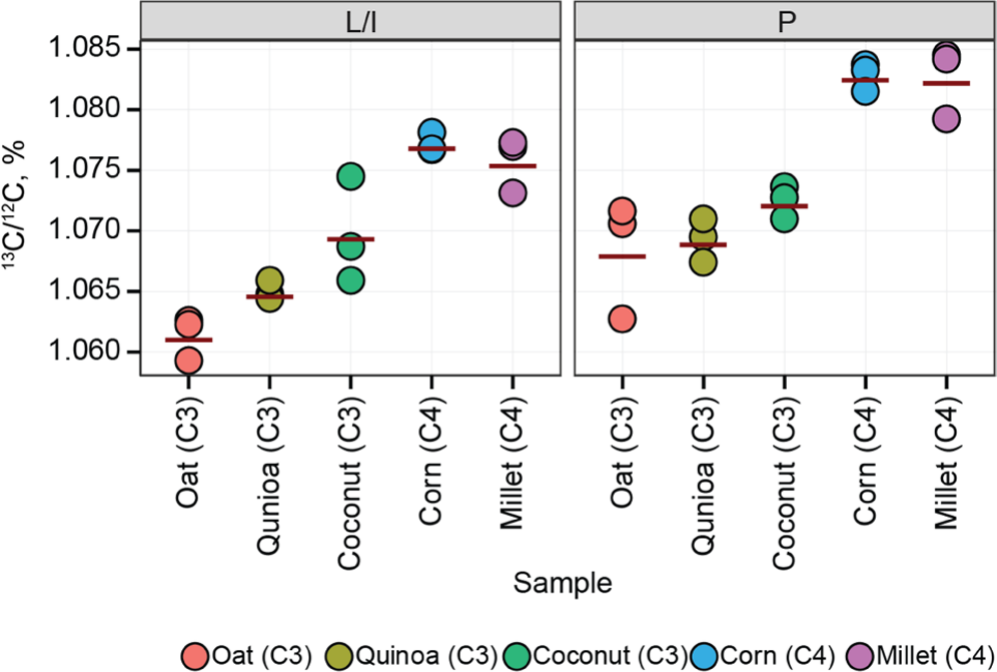
^13^C/^12^C ratios in Leu/Ile (L/I) and Pro (P) amino acid residues of proteins extracted from seeds of plants with C3 and C4 photosynthesis.

Plants using the C3 photosynthesis pathway are known to contain higher levels of ^13^C compared to rarer plants with C4-type photosynthesis,^31,32^ which provides good testing ground for LC-FT IsoR MS. Indeed, corn and millet samples (C4 plants) showed in FT IsoR MS higher ^13^C/^12^C ratios in both Leu/Ile and Pro compared to the C3 plants oat, quinoa, and coconut (Figure 4). Our FT IsoR MS in Pro showed δ^13^C of −47.8 ± 1.9‰ for C3 plants and −36.5 ± 0.14‰ for C4 plants. The δ^13^C difference between C3 and C4 types (11‰) in FT IsoR MS largely agrees with Gealy’s study (15‰).^32^

### LC-FT IsoR MS of Cell Lysates

**Figure 5:**
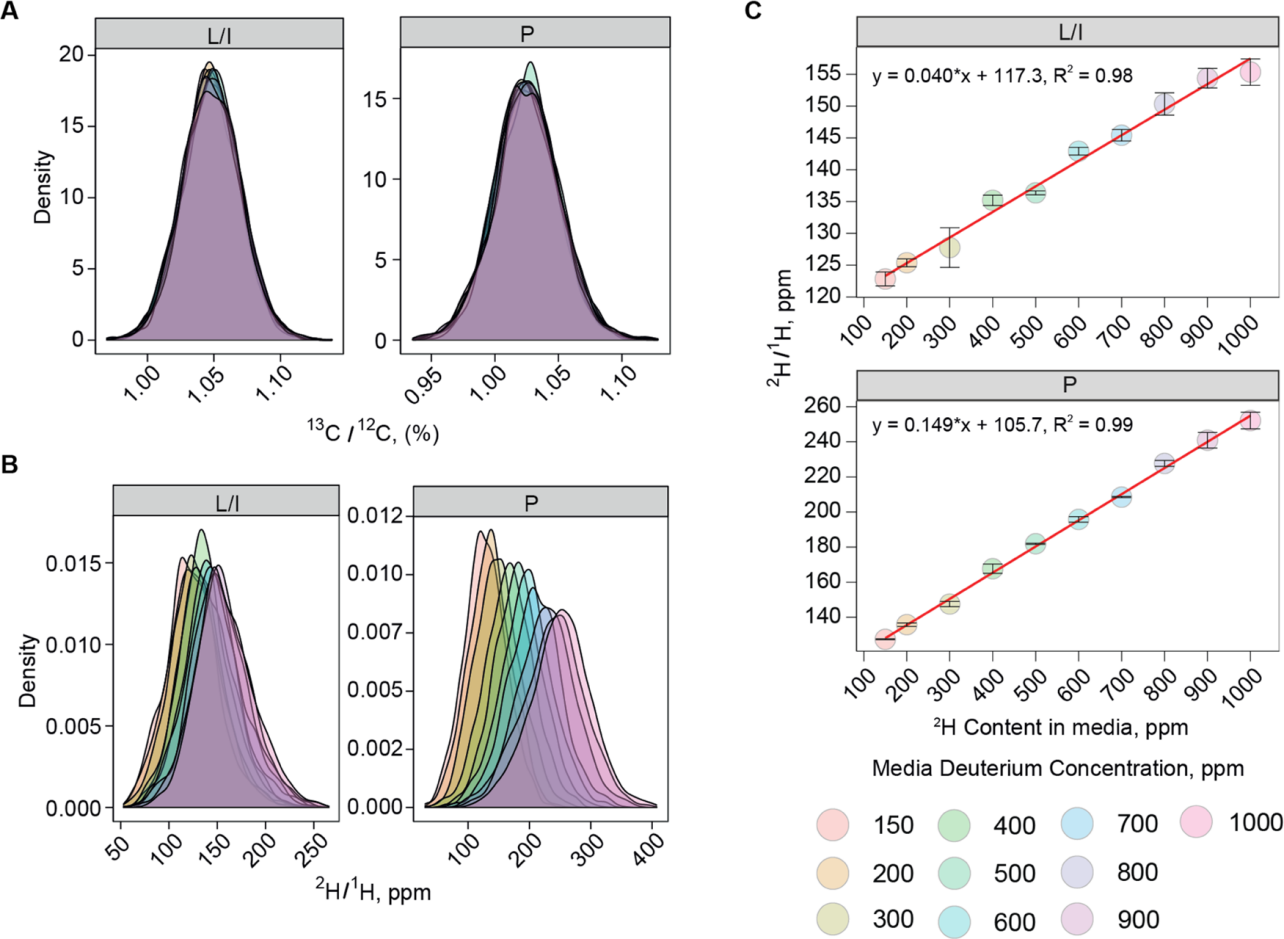
(A) Density distributions of ^13^C/^12^C ratios for two most abundant amino acids Leu/Ile (L/I) and Pro (P). (B) Density distributions of ^2^H/^1^H ratios for L/I and P. (C) Dependences between the measured ^2^H/^1^H ratios for L/I and P and the known ^2^H contents in the growth media.

Human fibroblast cell line was grown in media with 10 different deuterium concentrations ranging from 150 ppm ^2^H (control) to 1000 ppm ^2^H. As expected, the ^13^C/^12^C ratios were similar (no trend) for all of the samples and both analyzed immonium ions, Leu/Ile (isomers) and Pro (Figure 5A). On the other hand, the ^2^H/^1^H ratios changed linearly with the deuterium content in the media (Figure 5B). There is an almost perfect correlation (*R*^2^ > 0.98) between the FT IsoR MS readouts and the deuterium concentration in the media (Figure 5C). Based on the slope of linear regression, the incorporation degree of ^2^H was calculated to be (4.0 ± 0.3)% in Leu/Ile and (15 ± 1)% in Pro. The average CV between the replicates across the whole dataset was 0.3%.

### LC-FT IsoR MS of Bone Collagen

**Figure 6:**
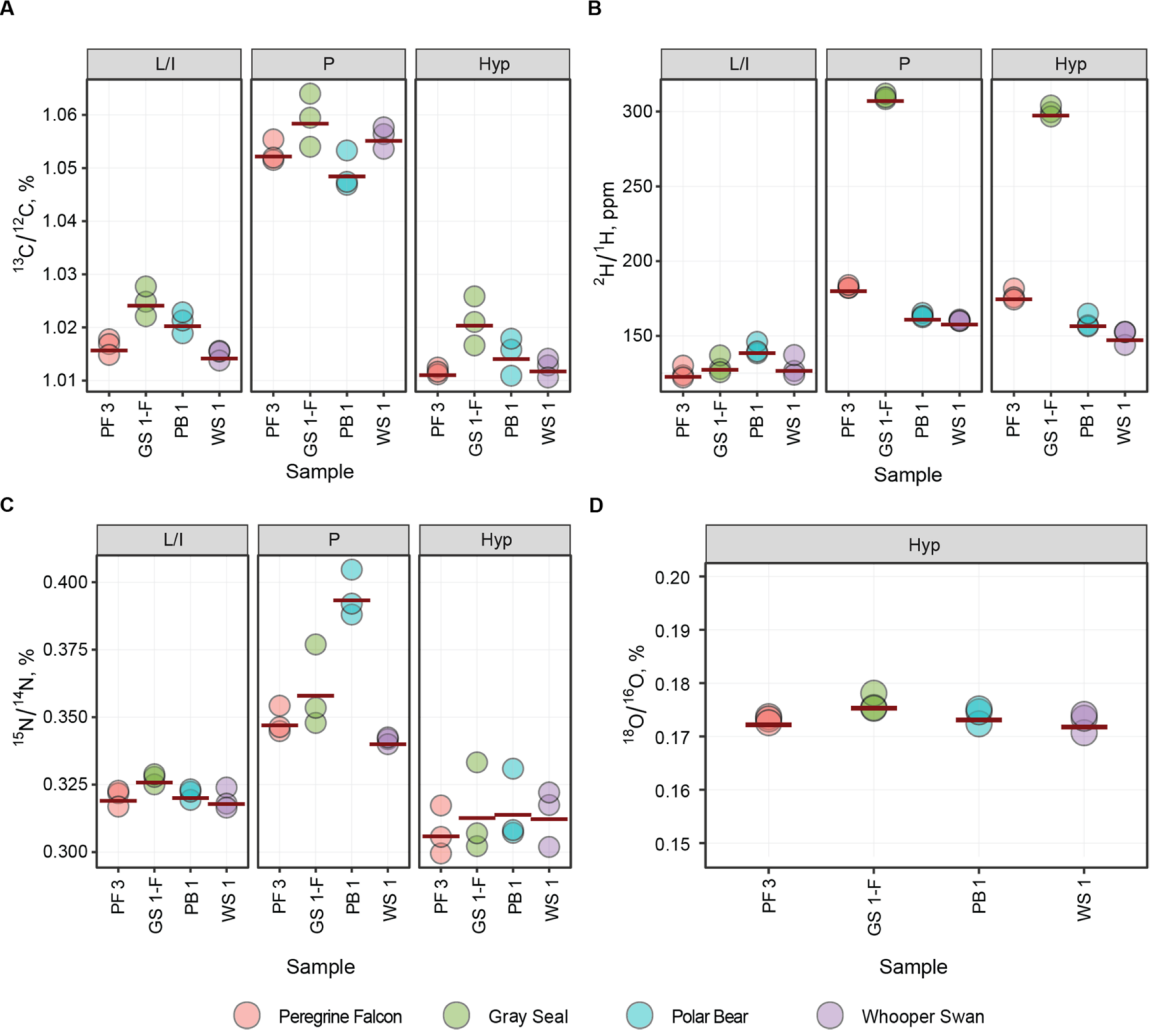
FT isoR MS isotopic ratio measurements of Leu/Ile, Pro, and Hyp in collagen samples for (A) ^13^C/^12^C, (B) ^2^H/^1^H, (C) ^15^N/^14^N, and (D) ^18^O/^16^O in peregrine falcon (PF3), gray seal (GS1-F), polar bear (PB1), and whooper swan (WS1).

Marine mammal collagen is known to contain higher ^2^H, ^13^C, and ^15^N amounts compared to terrestrial mammals.^31,33,34^ In the FT isoR MS analysis of several bone samples from different species, gray seal showed higher values for ^13^C in Pro, Leu/Ile, and hydroxyproline Hyp, the three abundant amino acids in collagen (Figure 6A). As we have recently shown, there is an abnormal amount of ^2^H in gray seal’s Pro and Hyp residues, exceeding the recorded ^2^H values in any previously analyzed living creature by ≈3-fold^26^ (Figure 6B). As expected,^35^ polar bear that is on a higher trophic level compared to gray seals showed a higher level of ^15^N enrichment in Pro, but surprisingly, there was no such difference in Hyp (Figure 6C). There was no difference in the ^18^O/^16^O ratios (Figure 6D), but on the other hand, only the Hyp immonium ion contains oxygen and was used for these measurements.

### LC-FT isoR MS of Isotopically Depleted *Escherichia coli*

One of the already mentioned advantages of FT IsoR MS is that the readout for abundant immonium ions is close to the final values, and thus, for measurements not requiring extreme accuracy, no comparison with well-characterized standards is required. This feature is especially convenient when the isotopic ratios deviate significantly from natural values and when no standards are available. To demonstrate this FT isoR MS feature, we grew *E. coli* on isotopically depleted media^36^ and analyzed extracted lysate proteins by FT isoR MS. The growth media were prepared using ^13^C depleted glucose, ^15^N depleted salt, and water with depleted ^2^H and ^18^O contents. As shown in Figure S2, more than 20 times depletion was detected for ^13^C/^12^C: 0.0441 ± 0.0008% [CV = 1.0%] in L/I and 0.0603 ± 0.0008% [CV = 0.7%] in P. For ^15^N/^14^N, almost 10-fold depletion was found, 0.0332 ± 0.0013% [CV = 2.3%] and 0.0555 ± 0.0015% [CV = 1.6%] for L/I and P, respectively. For ^2^H/^1^H, FT isoR MS showed 81.0 ± 0.5 ppm [CV = 0.3%] for L/I and 79.6 ± 1.2 ppm [CV = 0.9%] for P, which is consistent with most hydrogen in proteins originating from glucose rather than water.

### Number of Datapoints

**Figure 7:**
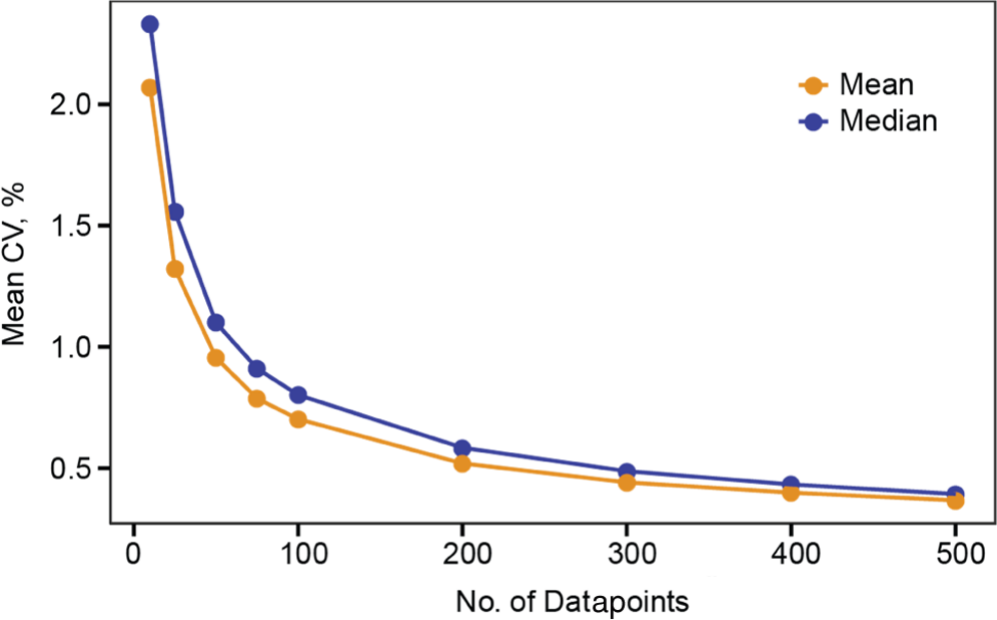
Average CV between the replicates for mean and median values of the isotopic ratio distributions for the elements C, N, and H in Ile/Leu and Pro in fibroblast lysate after randomly selecting different numbers of datapoints from the FT IsoR MS dataset.

The precision of FT IsoR MS is expected to depend upon the total ion charge Ne used for the measurements (e being the elementary charge), as the standard error of the mean (SEM) is proportional to N^–1/2^. Since AGC was employed, the number of ions in each mass spectrum was approximately the same, and thus, the precision should depend upon the number of spectra (datapoints) aggregated in the distribution shown in Figures 2 and 5A. Figure 7 depicts the dependence between the number of datapoints and precision of measurements (average CV between the replicates for C, N, and H isotopes). In analysis of fibroblast lysate, 100 points were sufficient for obtaining CVs below 1% (analysis time 5–10 min per replicate), and with 500 datapoints, the precision became <2‰ (analysis time 25–50 min).

### Attempt to Employ Internal Standard

Attempting to further improve the precision of FT IsoR MS measurements, we mixed two proteomes, A and B, with significantly different isotopic compositions and narrowed the *m*/*z* window to 2 *m*/*z* units in selecting the precursor ions for isotopic ratio measurements. Based on a database assignment of MS/MS spectra, each fragmented peptide was attributed to either proteome A or B, and the FT IsoR MS datasets were separated into sets A and B accordingly. While expecting to obtain two different sets of isotopic ratio values for proteomes A and B that should be close to the data obtained from individual FT IsoR MS analyses of the proteomes A and B, respectively, we observed two practically indistinguishable sets of isotopic ratios, with the average values in between those of A and B. We performed an identical experiment using this time field asymmetric ion mobility spectrometry (FAIMS), with very similar results. Narrowing the MS/MS selection window to 0.5 *m*/*z* units and limiting the dataset only to 100 most abundant peptides in each dataset yielded a qualitatively similar result.

### Species Verification

We searched CID = 35 MS/MS spectra from the FT IsoR MS dataset of seal collagen in the whole Swissprot database. Out of 69 proteins quantified using Proteome Discoverer software, 34 proteins were collagenous proteins. The most abundant collagen was from dog (*Canis lupus*), the closest to seal species in the Swissprot database (the same *Carnivora* order). In general, the Swissprot database lacked collagen sequences from any of the species presented in this study, which calls for creation of a special collagen sequence database.

Another parameter that could be used for verifying the sample identity from FT isoR MS data is the relative abundance of amino acids. For instance, Pro, Hyp, Leu/Ile, and Phe contribute to gray seal collagen data (Figure 5) 35, 22, 9, and 5%, respectively, which agree rather well (*R*^2^ = 0.8) with the known average values for collagen: 18% for Pro, 11% for Hyp, 7% for Leu + Ile, and 5% for Phe.^37^

## Discussion

Here, we investigated in detail the FT isoR MS performance in isotopic ratio analysis for C, H, and N and demonstrated it for O. The analysis is performed with simultaneous verification of the protein sequence and composition of most abundant amino acids. In each FT isoR MS analysis, only 1 μg of protein digest was used, which is 3 orders of magnitude below the standard IsoR MS requirements. Reliable and consistent FT IsoR MS measurements could be obtained as long as there are ≥100 datapoints per sample available. The CVs between the replicate analyses were usually in low per mil regions for C and N isotopic measurements and below 10‰ for H. Within the AGC operational range, there was no discernible dependence detected of the FT IsoR MS data upon the ion current, which gives hope that the sample consumption can be further reduced. The fact that the chosen FT IsoR MS approach to data acquisition (Figure 3A) provided data repeatable on a month-long time scale gives certainty that good accuracy can be achieved using proper standards.

Unfortunately, the attempt to improve the accuracy of measurements by employing one proteome as an internal standard for the other proteome was unsuccessful. We attributed this failure to the presence of very abundant background, invisible in the FT mass spectra, composed of clusters of molecules from both proteomes.

Compared to CSIA, the FT isoR MS approach is advantageous due to its sensitivity and the absence of derivatization. Also, when modest accuracy is sufficient, no standards are required, which is convenient in characterization of samples with strongly deviating isotopic composition.

One current limitation of the FT IsoR MS implementation on most commercial Orbitrap instruments is the lowest detectable *m*/*z* value of 50, which is higher than *m*/*z* values for two most abundant amino acids in collagen, Gly and Ala. While newer Orbitrap instruments have the lowest *m*/*z* 40, to overcome this problem using older instrumentation, acid hydrolysis of protein may be implemented to release free amino acids (MH+ ion of Gly has *m*/*z* 76, and for Ala, it is 90), at the expense of losing the accuracy of hydrogen isotope analysis. Another limitation is the absence of distinction between leucine and isoleucine residues in immonium ion analysis. However, these two amino acids in a free state are easily separated by liquid chromatography, and thus, LC-isolated free amino acids can be individually analyzed by FT isoR MS. When implementing FT IsoR MS in a proteomics lab, it is important to ensure the absence of interferences; for instance, traces of tandem mass tag (TMT) reagents can affect isotopic measurements in proline.

## Conclusions

Our Orbitrap-based FT isoR MS analysis can simultaneously provide isotopic ratio data and verify the protein amino acid composition and sequence in 1 μg protein digest. The accuracy of this method can be further improved by developing proper standards.

## Supporting Information Available

Additional details on data analysis, such as peak picking algorithm and results from different experiments including different MS type events and plots for *E. coli* isotopic distribution (PDF)
