# Supplementary material for: Proteomics-Compatible Fourier Transform Isotopic Ratio Mass Spectrometry of Polypeptides
Source: Anal Chem. 2022 Oct 17;94(43):15048–56. doi: 10.1021/acs.analchem.2c03119 (PMC9631351; doi:10.1021/acs.analchem.2c03119)
Supplement: Supplementary file 1 — ac2c03119_si_001.pdf [file ac2c03119_si_001.pdf]

Supporting Information for

Manuscript title: Proteomics-compatible Fourier Transform Isotopic Ratio Mass Spectrometry of Polypeptides

Hassan Gharibi[1‡], Alexey L. Chernobrovkin[2‡], Amir Ata Saei[1,3§], Xuepei Zhang[1,4,5], Massimiliano Gaetani[1,4,5], Alexander A. Makarov[6], Roman A. Zubarev[1,7,8*]

[1]Division of Physiological Chemistry I, Department of Medical Biochemistry and Biophysics, Karolinska Institutet, Stockholm, Sweden, SE 171 77

[2]Pelago Biosciences, Solna, Sweden, SE 171 65

[3]Department of Cell Biology, Harvard Medical School, Boston, U.S., MA 02115

[4]Chemical Proteomics, Department of Medical Biochemistry and Biophysics, Karolinska Institutet, Stockholm, Sweden, SE 171 77

[5]Unit of Chemical Proteomics, Science for Life Laboratory (SciLifeLab), Stockholm, Sweden, SE 171 77

[6]Thermo Fisher Scientific GmbH, Bremen, Germany, DE 28199

[7]Department of Pharmacological & Technological Chemistry, I.M. Sechenov First Moscow State Medical University, Moscow, 119146, Russia

[8] The National Medical Research Center for Endocrinology, 115478 Moscow, Russia

[§]Current address: Biozentrum, University of Basel, 4056 Basel, Switzerland

Table of content

Data Analysis 3

Peak Picking Algorithm 3

The Gaussian Curve Fitting 3

Figures 4

Data Analysis

Peak Picking Algorithm

To find a peak from data acquired in profile mode, first we created a list for the masses of immonium ion fragments of all the amino acids, containing the number of each element in them. Then we defined a mass tolerance window for searching these masses (m/z), accounting for the mass shift that could happen in mass spectrum result (0.0015 Da). Using the monoisotopic masses and the mentioned window, we then search each spectrum for the corresponding m/z of individual immonium ion fragments. We defined the center m/z via finding the most intense point (aka apex). The mass-error was calculated based on the difference between the center m/z and theoretical m/z. To consider the founded points as a good peak, each peak needed to be consisted of at least 10 data points.

The Gaussian Curve Fitting

Once the datapoints for individual peak is found, we fit a gaussian function using a set of parameters. Gaussian function is displayed below:

$$f(x) = Ae^{-\frac{(x-mu)^2}{2\sigma^2}}$$

Where A is the height of the gaussian fit, mu is the center point (or the mean value), and σ is the standard deviation.

The initial parameters for the gaussian fit are as followed:

A = Intensity of the highest point (apex)
mu = m/z of the apex point
σ = standard deviation of the m/z where we defined the monoisotopic peak m/z range

We then compared the fitted gaussian model to the recorded values and if the fit has a good R^2 (higher than 0.9 for monoisotopic peak and 0.5 for isotopic peak), then we calculate the area under the curve of the gaussian model took it as the representative of the peak abundance. Later, we filtered out any measurement with absolute mass-error higher than 3×10^{-4} m/z.

Figures

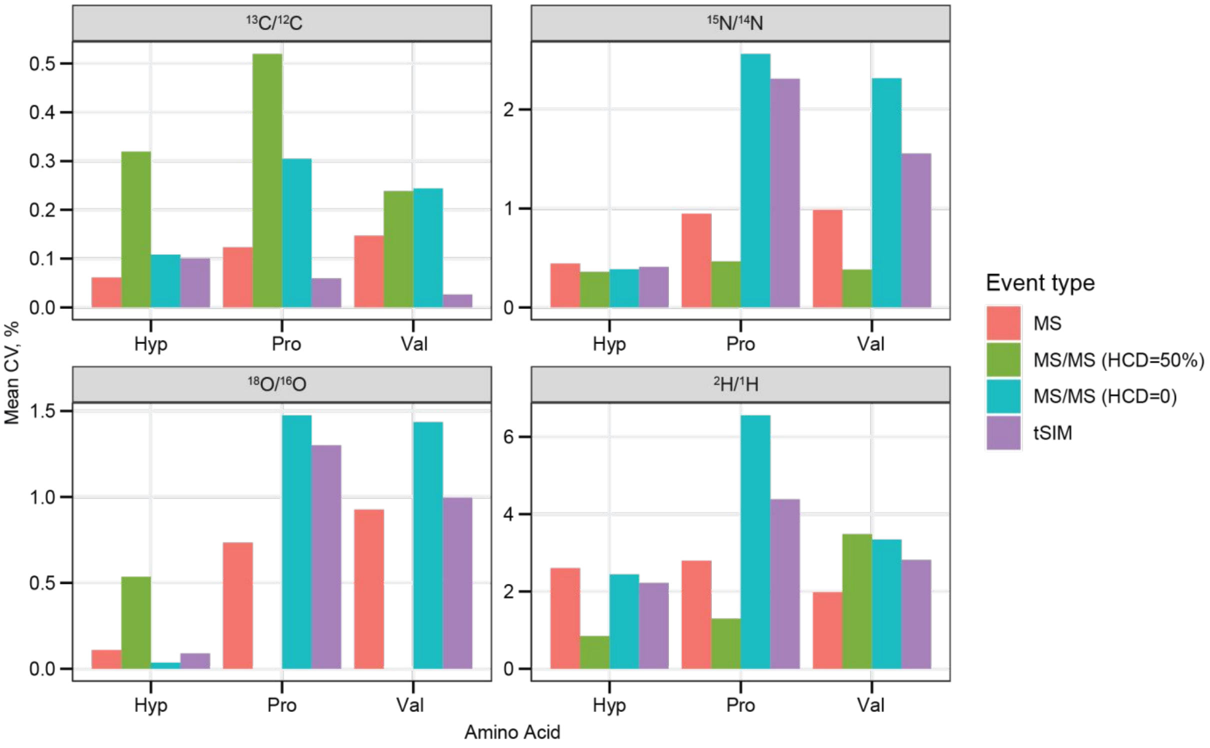

Figure S1. Average CVs between the replicates for different specific MS and MS/MS event types.

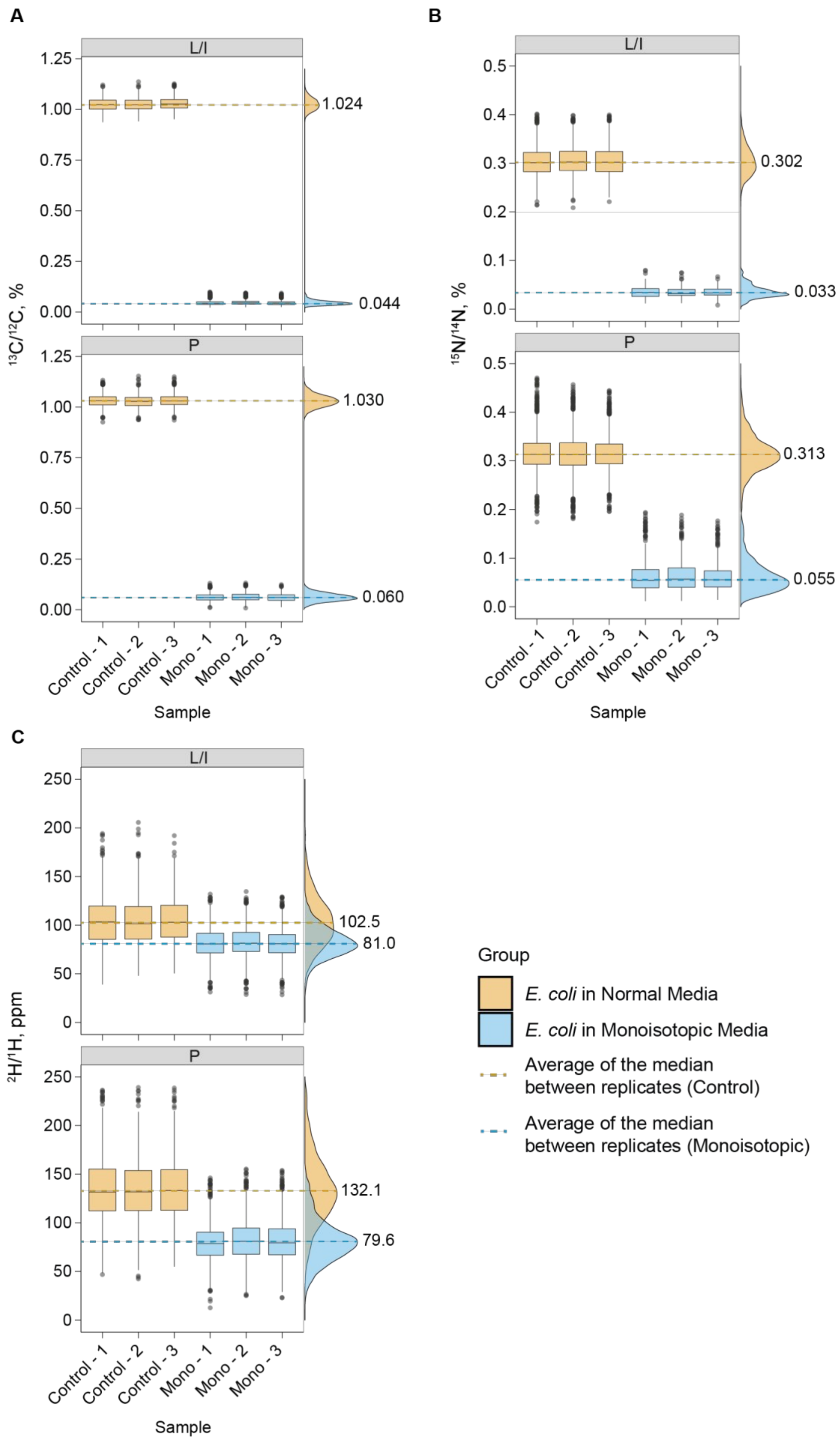

Figure S2. LC-FT isoR MS readouts of *E. coli* grown on isotopically depleted media for $^{13}C/^{12}C$ (A), $^{15}N/^{14}N$ (B), and $^{2}H/^{1}H$ (C) (n = 3).
